# Impact of COVID-19 on ophthalmic surgical procedures in sub-Saharan Africa: a multicentre study

**DOI:** 10.1186/s41182-024-00589-1

**Published:** 2024-03-15

**Authors:** Naseer Ally, Sarah Ismail, Natasha Naidu, Ismail Makda, Ismail Mayet, Michael E. Gyasi, Peter Makafui, Arlette Nomo, Chantal Nanfack, Anesu T. Madikane, Walda D. Pohl, Bayanda N. Mbambisa, Jonathan T. Oettle, Feyi Adepoju, Toibat B. Tota-Bolarinwa, Amelia Buque, Sidonia J. N. Khalau, Douglas Zirima, Brian Takayidza, Ugochukwu A. Eze, Akinyemi Adedeji, Frank Sandi, Jacinta Feksi, Ogugua Okonkwo, Adekunle Hassan, Nagib du Toit, Shahlaa Petersen, Caroline Tsimi, Viola Dovoma, Mustapha Bature, Mohammed Adamu, Suhanyah Okeke, Ifeoma N. Asimadu, Nkiru N. Kizor-Akaraiwe, Chinyelu N. Ezisi, Henry E. Nkumbe, Tchoyou T. M. Olivier, Hassan D. Alli

**Affiliations:** 1 St John Eye Hospital, University of the Witwatersrand, Johannesburg, South Africa; 2 St Thomas Eye Hospital, Accra, Ghana; 3 Yaounde Gynaeco-Obstetric and Paediatric Hospital, Yaounde, Cameroon; 4 Tshwane District Hospital, Tshwane, South Africa; 5 Livingstone Hospital, Walter Sisulu University, Gqeberha, South Africa; 6 University of Ilorin Teaching Hospital, Ilorin, Nigeria; 7 Dr Agarwal’s Eye Hospital, Maputo, Mozambique; 8 Sekuru Kaguvi Eye Unit, Paririnyetwa Hospital, Harare, Zimbabwe; 9 Federal Medical Centre, Asaba, Nigeria; 10 The University of Dodoma Medical School, Dodoma, Tanzania; 11 Eye Foundations Hospital, Lagos, Nigeria; 12 Groote Schuur Hospital, University of Cape Town, Cape Town, South Africa; 13 Yaounde Central Hospital, Yaounde, Cameroon; 14 Usmanu Danfodiyo University Hospital, Sokoto, Nigeria; 15 Enugu State University Teaching Hospital, Enugu, Nigeria; 16 The Eye Specialists Hospital, Enugu, Nigeria; 17 Magrabi ICO Cameroon Eye Institute, Yaounde, Cameroon

**Keywords:** COVID-19, Ophthalmology, Sub-Saharan Africa, Ophthalmic surgery, Cataract, Glaucoma, Cornea, Vitreoretinal, Trauma, Oculoplastic

## Abstract

**Background:**

The COVID-19 pandemic had a profound impact on healthcare and ophthalmology services globally. Numerous studies amongst various medical and surgical specialties showed a reduction in patient attendance and surgical procedures performed. Prior published ophthalmic literature focused on specific types of procedures and were usually single centre. The current study attempts to quantify the impact on a larger scale, namely that of sub-Saharan Africa, and to include all ophthalmic subspecialties.

**Methods:**

This is a retrospective analysis of the surgical records from 17 ophthalmology centres in seven countries located in East, Central, West and Southern Africa. The date of declaration of the first lockdown was used as the beginning of the pandemic and the pivot point to compare theatre records one year prior to the pandemic and the first year of the pandemic. We examined the total number of surgical procedures over the two year period and categorized them according to ophthalmic subspecialty and type of procedure performed. We then compared the pre-pandemic and pandemic surgical numbers over the two year period.

**Results:**

There were 26,357 ophthalmic surgical procedures performed with a significant decrease in the first year of the pandemic (*n* = 8942) compared to the year prior to the pandemic (*n* = 17,415). The number of surgical procedures performed was lower in the first year of the pandemic compared to the year prior to the pandemic by 49% [Incidence rate ratio (IRR) 0.51, 95% CI 0.41–0.64), 27% (0.73, 0.55–0.99), 46% (0.54, 0.30–0.99), 40% (0.60, 0.39–0.92) and 59% (0.41, 0.29–0.57) in sub-Saharan Africa (4 regions combined), West, Central, East and Southern Africa, respectively]. The number of surgical procedures in the different sub-specialty categories in sub-Saharan Africa (4 regions combined) was significantly lower in the first year of the pandemic compared to the year prior to the pandemic, except for glaucoma (IRR 0.72, 95% CI 0.52–1.01), oncology (0.71, 0.48–1.05), trauma (0.90, 0.63–1.28) and vitreoretinal (0.67, 0.42–1.08) categories.

**Conclusion:**

This study provides insight into the impact of the COVID-19 pandemic in multiple regions and countries on the African continent. The identification of which surgical subspecialty was most affected by the COVID-19 pandemic in each region allows for better planning and resource allocation to address these backlogs.

## Introduction

Globally, almost 768 million confirmed cases of COVID-19, including 6.9 million deaths have been reported [1]. Approximately, 9.5 million confirmed cases have been reported in Africa. Since the pandemic, healthcare services worldwide have been overburdened by the backlog of medical appointments and elective surgical cases [2–5]. This backlog is due to the cancellation or delay of non-urgent appointments [4, 6], ill healthcare workers [5], and the redirection of hospital resources to acute care at the height of the pandemic [6]. An analyses of data from 228 hospitals in 40 states revealed a 54% decrease in the number of patients accessing healthcare in the United States from March 24 to April 6, 2020 during the pandemic; the largest decrease in patient volume was demonstrated by ophthalmology (81% compared to a similar period in 2019) [7].

To accommodate the increased burden of COVID-19 cases on hospital services during the pandemic, elective surgical procedures [8, 9], including ophthalmic surgical procedures [10–12], were reduced. A single-centre study in the USA, reporting on different ophthalmic surgical procedures, observed an 89% reduction in elective surgical procedures during the COVID-19 pandemic compared to a similar period before the pandemic [12]. Whereas the most common surgical procedure before the pandemic was elective cataract surgery (47.3% of all ocular surgical procedures performed), the most common surgical procedure during the pandemic was acute retinal detachment repair (31.6%). A similar study in the UK, comparing corneal surgical procedures during the pandemic with a similar period before the pandemic, noted a 92% decrease in elective surgical cases, but an increase in acute traumatic corneal repairs from 1 to 9 cases [11]. A large multi-centre study in India reported a 94.4% decrease in cataract surgical cases during the pandemic [13]. Recent studies in Nigeria and the United Kingdom reported an increase in the number of ocular trauma cases during the pandemic as opposed to pre-pandemic [14, 15]. In contrast, studies from India and the USA reported a decline in ocular trauma during the COVID-19 pandemic when compared to the year prior to the pandemic [16, 17].

Whilst some studies have reported the impact of the pandemic within institutions and on specific individual categories of ophthalmic surgical procedures [11–13], there have been, to our knowledge, no studies to date that have quantified the impact of the initial year of the pandemic over larger geographic areas or spanning multiple countries within a particular continent. The current study aimed to quantify the initial impact the COVID-19 pandemic had during the first year on various ophthalmic surgical procedures in sub-Saharan Africa, including centres in East, Central, West and Southern Africa.

## Methods

This is a retrospective analysis of the surgical records from 17 ophthalmology centres in seven countries located in East, Central, West and Southern Africa (Table 1). We used the date of declaration of the lockdown as the start of the pandemic and as the pivot point to compare theatre records one year prior to the pandemic and one year after the pandemic began. The exact dates of the pandemic, lockdown and waves are shown in Appendix A. These were defined as the year prior to the pandemic and the first year of the pandemic, respectively. Although all countries had a two year period that was assessed (one year pre-pandemic and the first year of the pandemic) the exact dates differed as these were different across the continent. The first year of the pandemic was chosen as this was when the impact of the COVID-19 pandemic was greatest. The study was coordinated by the investigators from St John Eye hospital, University of the Witwatersrand, Johannesburg, South Africa. We obtained ethical approval at this centre from the Human Research Ethics committee and adhered to the tenets of the declaration of Helsinki. Each study site was responsible for obtaining ethical approval from the relevant local Institutional Review Board/Ethics committee. Some sites were granted a waiver by their local ethic committee/IRB due to the retrospective nature of the study or accepted the ethics approval from the coordinating centre. The remaining institutions obtained independent ethical/IRB approval.

**Table 1:** Participating centres in sub-Saharan Africa with surgical sub-specialty types

Hospital	Cataract	Cornea	Glaucoma	Oncology	Orbital/Oculoplastic	Strabismus	Trauma	Vitreoretinal
**West Africa**								
St Thomas Eye Hospital (Accra, Ghana)	X	X	X	X		X		X
Federal Medical Centre (Asaba, Nigeria)	X	X	X	X	X	X	X	
Enugu State University Teaching Hospital (Enugu, Nigeria)	X	X	X	X	X		X	
Eye Foundations Hospital (Lagos, Nigeria)	X	X	X		X	X	X	X
The Eye Specialists Hospital (Enugu, Nigeria)	X	X	X	X	X	X	X	
University of Ilorin Teaching Hospital (Ilorin, Nigeria)	X		X	X	X	X		
Usmanu Danfodiyo University Hospital (Sekoto, Nigeria)	X	X	X	X	X		X	
**Central Africa**								
Yaounde Gynaeco-obstetric and paediatric hospital (Yaounde, Cameroon)	X	X	X	X	X	X	X	
Yaounde Central Hospital (Yaounde, Cameroon)	X		X	X	X	X	X	
Magrabi Eye Institute (Yaounde, Cameroon)	X	X	X	X	X	X	X	X
**East Africa**								
Benjamin Mkapa Hospital (Dodoma, Tanzania)	X	X	X	X	X		X	
**Southern Africa**								
St John Eye Hospital, University of the Witwatersrand (Johannesburg, South Africa)	X	X	X	X	X	X	X	X
Sekuru Kaguvi Eye Unit, Paririnyetwa Hospital (Harare, Zimbabwe)	X	X	X	X	X	X	X	X
Tshwane District Hospital (Tshwane, South Africa)	X	X	X	X	X			
Groote Schuur Hospital, University of Cape Town (Cape Town, South Africa)	X	X	X	X	X	X	X	X
Port Elizabeth Provincial Hospital (Gqeberha, South Africa)	X	X	X	X	X	X	X	X
Dr Agarwal’s Eye Hospital (Maputo, Mozambique)	X	X	X	X	X	X		X

X indicates the surgical subspecialty types offered at each hospital

Three hospitals had digital records, namely, Dr. Agarwal’s Eye Hospital, Maputo, Mozambique; Groote Schuur Hospital, Cape Town, South Africa; and St. Thomas Eye Hospital, Accra, Ghana. The remaining centres had written records available. We examined and collected the following data from theatre records over the two year period: date of lockdown in the country and number of surgical procedures performed in each of the two years according to the ophthalmic sub-specialty category. The date of the lockdown served as the beginning of the COVID-19 pandemic year and the year prior to that was taken for comparison. The dates of the lockdown, and months of the waves are detailed in Appendix A. The sub-specialty categories included cataract, cornea, glaucoma, oncology, orbital and oculoplastic, vitreoretinal, strabismus and trauma. Adult and paediatric surgical procedures were combined in each sub-specialty category. Cataract surgical procedures included phacoemulsification, manual small incision cataract surgery (MSICS), extra capsular lens extraction (ECLE) and paediatric lens washout (LWO); corneal surgeries included penetrating keratoplasty (PK), deep anterior lamellar keratoplasty (DALK), descemet stripping endothelial keratoplasty (DSEK) and descemet’s membrane endothelial keratoplasty (DMEK); glaucoma included glaucoma tube shunts, trabeculectomies, trabeculotomies and goniotomies; oncology included enucleations and exenterations for tumours; orbital and oculoplastic included orbital decompressions and lid surgeries, vitreoretinal included pars plana vitrectomies and scleral buckling procedures; strabismus included extraocular muscle recessions/resections; trauma included corneal and scleral laceration repairs and eviscerations for globe ruptures. Non-specific surgical procedures which did not fit in any of the above categories were classified as “Other”.

Each centre had two investigators, one who collected and populated the data collection sheet from the theatre records and a second who checked and verified the data. The data were then sent to the coordinating site (University of the Witwatersrand) and underwent a second verification by the coordinating site. Any queries that arose at the coordinating site were addressed to the primary site and resolved through discussion.

### Statistical analysis

We analysed the data using Stata 16.1 (STATACorp, College Station, Texas). Crude analysis of the counts of each surgical sub-specialty at each institution was analysed using a one sample proportions test over the two years. The null hypothesis was that there is no difference between the pre-pandemic and pandemic proportions. This meant that each period should contribute a 50% proportion of surgeries in using the one sample proportions test. Univariate and multivariate negative binomial regression models were then used to quantify the effect of the pandemic (the entire year), and waves (first and second) on the number of surgical procedures performed according to each region and subspecialty. The multivariate analysis compared the pre-pandemic year and the first year of the pandemic, whilst adjusting for the waves during the pandemic to ascertain whether there was a further change in surgical numbers over and above the reduction caused by the pandemic itself. The regression coefficients were reported as Incidence Rate Ratios (IRR). Graphs for all regions were then generated by overlaying the scatterplots of the data with local polynomial smoothing for the number of surgeries per month over the 24-month period. A median spline was generated for East Africa. A *p* value of < 0.05 was regarded as significant.

## Results

Seventeen hospitals in four designated regions of East (1), West (7), Central (3) and Southern Africa (6) participated in the study (Table 1). The only hospital from East Africa that participated was in Tanzania. The West African hospitals comprised six hospitals from Nigeria and one hospital from Ghana. In the Central African region, all three hospitals were from Cameroon. The Southern African hospitals comprised four hospitals from South Africa, one from Zimbabwe and one from Mozambique. The breakdown of the hospitals in each region, including the surgical sub-specialty types offered, is shown in Table 1.

**Fig. 1:**
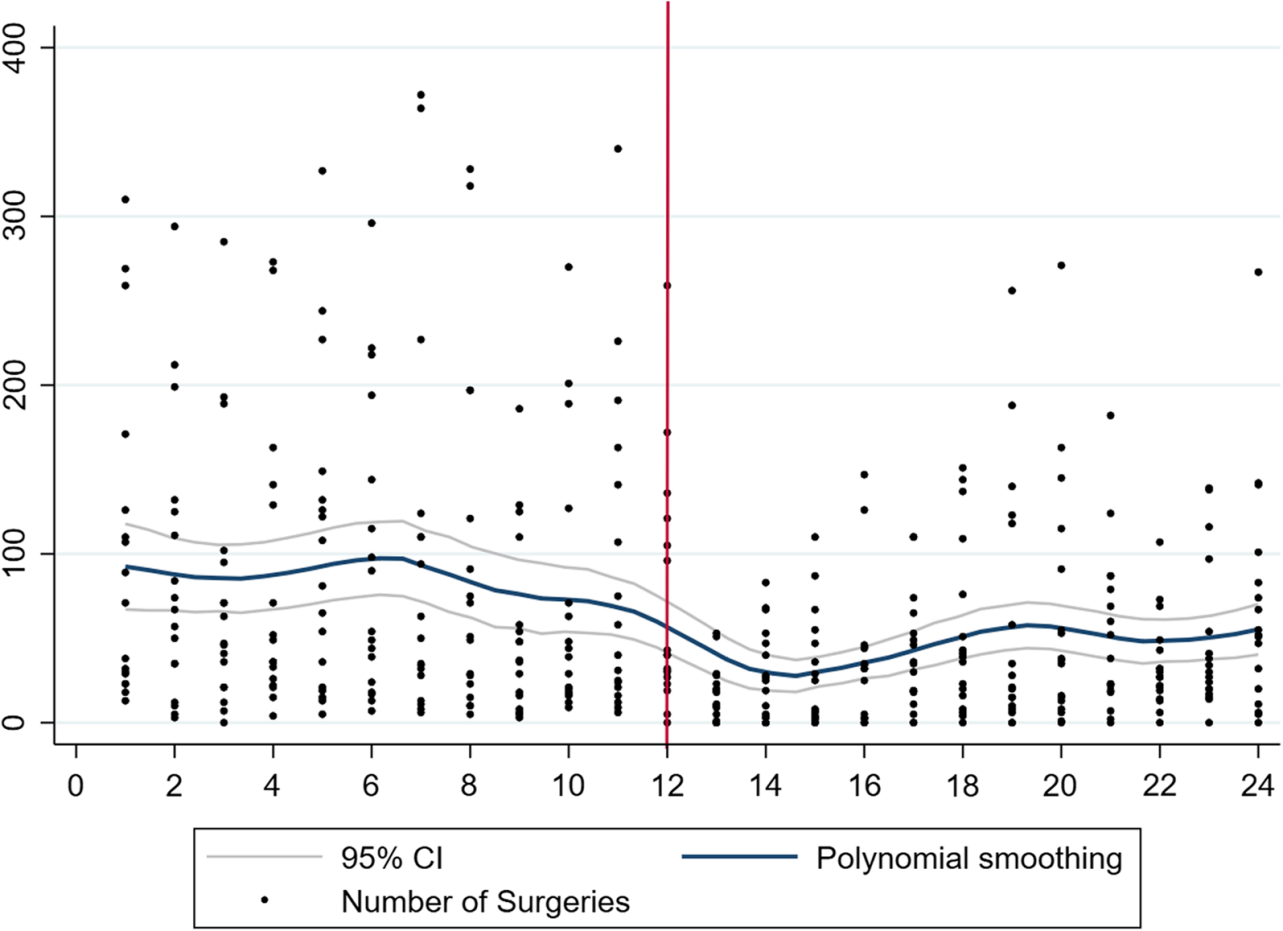
Scatterplot with polynomial smoothing of number of surgical procedures in sub-Saharan Africa with 95% confidence interval

**Fig. 2:**
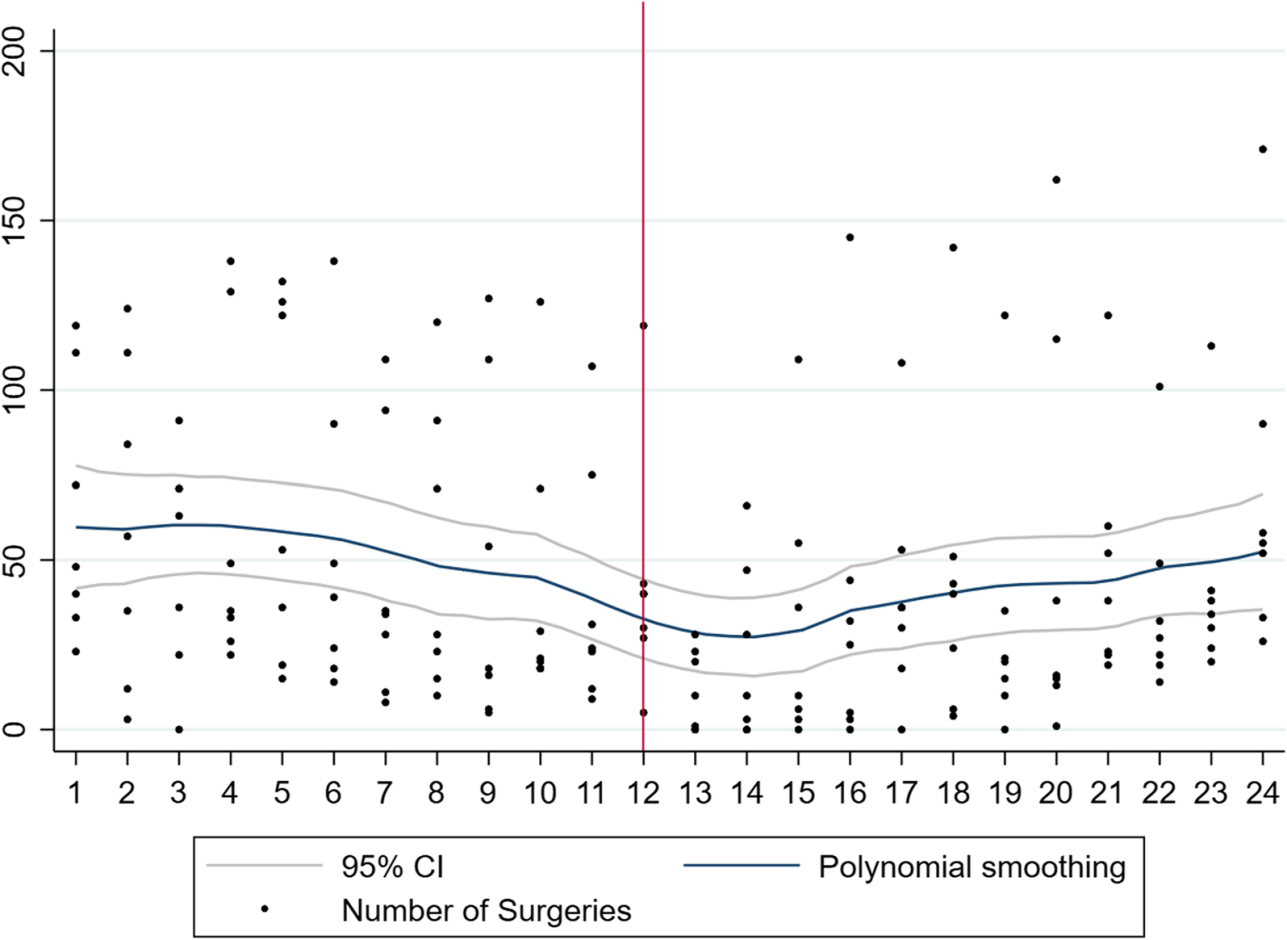
Scatterplot with polynomial smoothing of number of surgical procedures in West Africa with 95% confidence interval

**Fig. 3:**
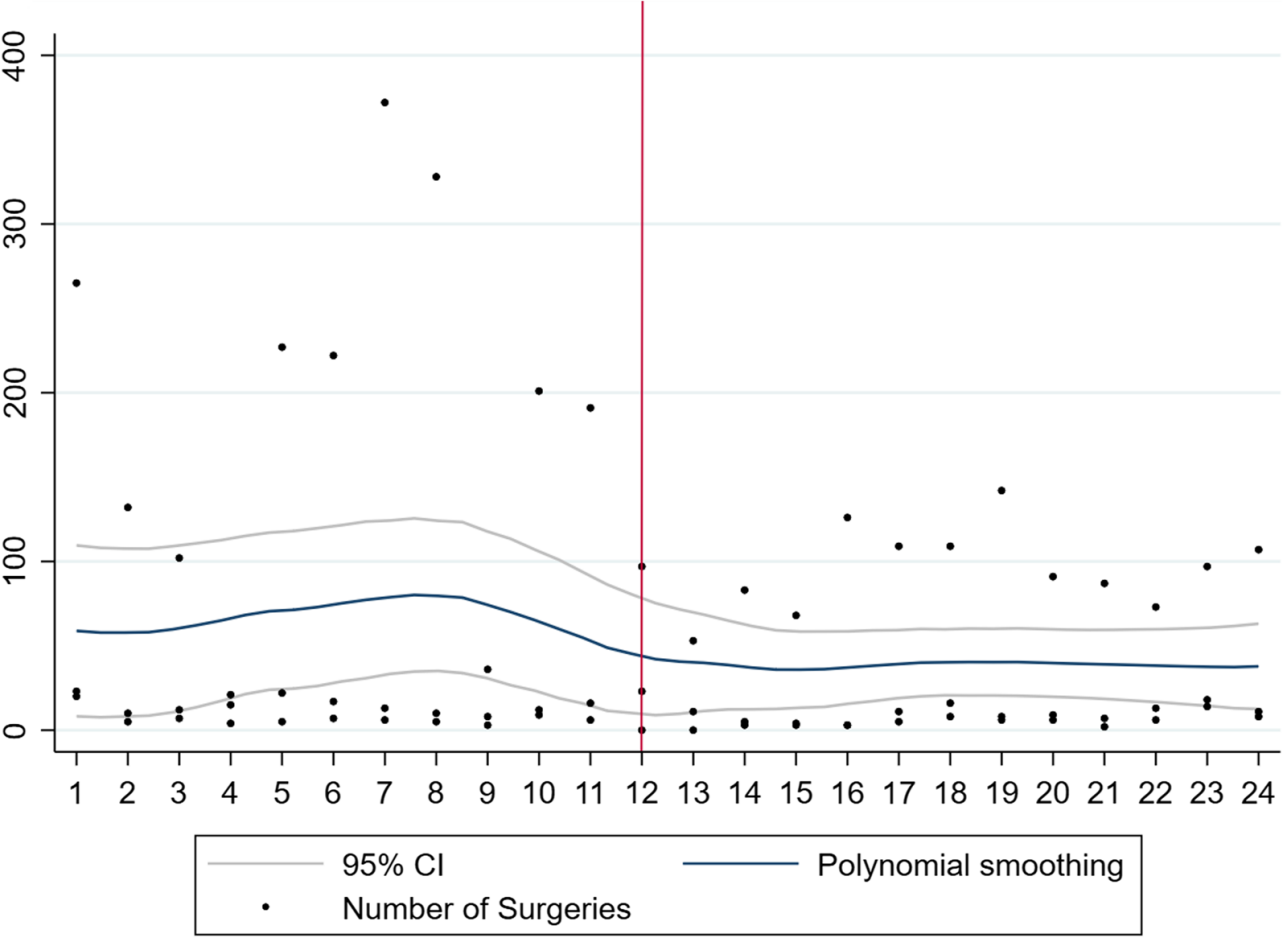
Scatterplot with polynomial smoothing of number of surgical procedures in Central Africa with 95% confidence interval

**Fig. 4:**
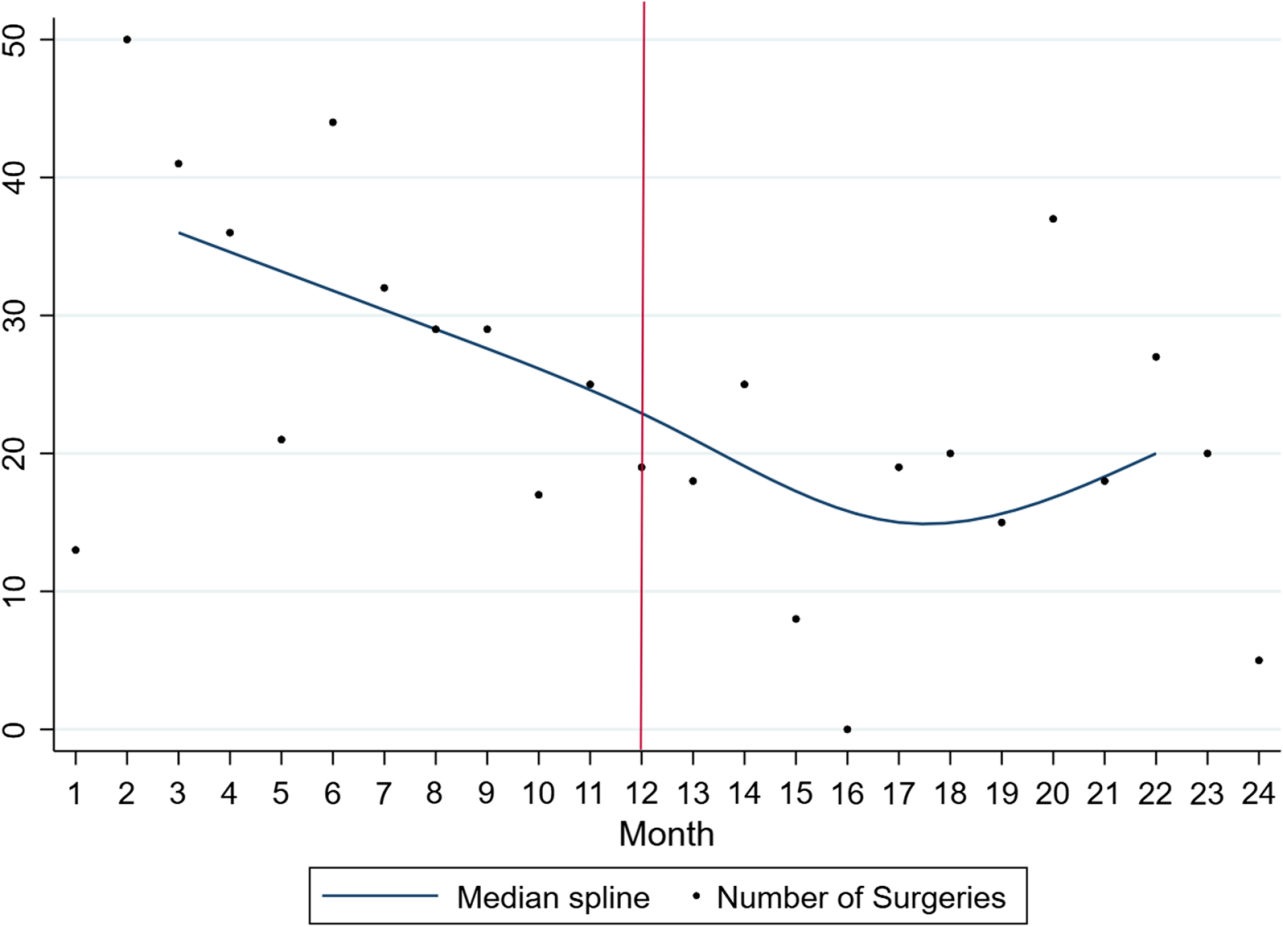
Median spline of number of surgeries in East Africa

**Fig. 5:**
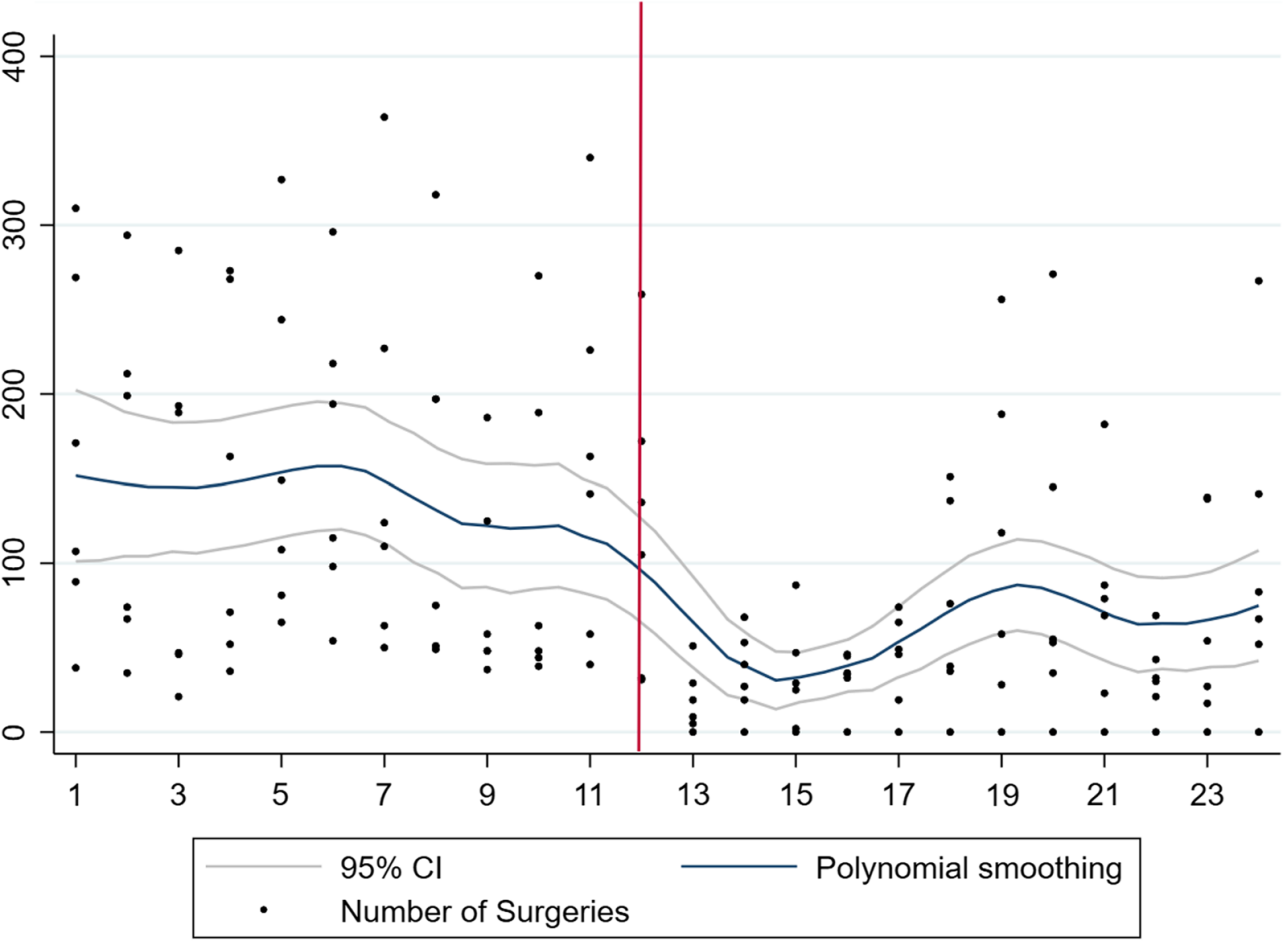
Scatterplot with polynomial smoothing of number of surgical procedures in Southern Africa with 95% confidence interval

Overall, there were 26,357 ophthalmic surgical procedures performed with a significant decrease in the first year of the pandemic (*n* = 8942) compared to the year prior to the pandemic (*n* = 17,415). Also, there was a significant reduction in procedures performed in each of the four regions, East (353 to 212), Central (2452 to 1325), West (4433 to 3241), and Southern Africa (10,177 to 4137). Graphically, the total number of surgical procedures in sub-Saharan Africa, West Africa, Central Africa, East Africa, and Southern Africa are represented in Figs. 1, 2, 3, 4, and 5, respectively. All centres had a significant decline in the number of surgical procedures except for two centres, one in West Africa (Nigeria) and one in Central Africa (Cameroon) (Table 2).

**Table 2:** Total number of surgical procedures performed over the two year period with the pre-pandemic and pandemic numbers included

Region	Total number of surgeries *n*	Pre-pandemic number of surgical procedures *n*, % of total (95%CI)	Pandemic number of surgical procedures *n*, % of total (95%CI)	*p* value^a^
**Sub-Saharan Africa**	26 357	17 415 (66.1) [65.5–66.7]	8942 (33.9) [33.4–34.5]	< 0.001
West Africa	7701	4433 (57.6) [56.5–58.7]	3241 (40.0) [40.9–43.2]	< 0.001
St Thomas Eye Hospital (Accra, Ghana)	1565	1063 (67.5) [65.6–70.2]	502 (22.5) [29.8–34.4]	< 0.001
Federal Medical Centre (Asaba, Nigeria)	388	222 (57.2) [52.3–62.1]	166 (21.8) [37.9–47.7]	0.005
Enugu State University Teaching Hospital (Enugu, Nigeria)	661	500 (75.6) [72.4–78.9]	161 (24.4) [21.1–27.6]	< 0.001
Eye Foundations Hospital (Lagos, Nigeria)	2816	1432 (50.1) [49.0–52.7]	1384 (49.9) [47.3–51.0]	0.366
The Eye Specialists Hospital (Enugu, Nigeria)	756	335 (44.3) [40.8–47.9]	421 (55.7) [52.1–59.2]	0.002
University of Ilorin Teaching Hospital (Ilorin, Nigeria)	1132	721 (63.7) [60.9–66.5]	411 (36.3) [33.5–39.1]	< 0.001
Usmanu Danfodiyo University Hospital (Sokoto, Nigeria)	383	160 (41.8) [36.8–46.7]	223 (58.2) [53.3–63.2]	0.001
**Central Africa**	3777	2452 (64.9) [63.4–66.4]	1325 (35.1) [33.6–35.6]	< 0.001
Yaounde Gynaeco-obstetric and paediatric hospital (Yaounde, Cameroon)	172	93 (54.1) [46.6–61.5]	79 (45.9) [38.5–53.4]	0.286
Yaounde Central Hospital (Yaounde, Cameroon)	266	165 (62.0) [56.2–67.9]	101 (38.0) [32.1–43.8]	< 0.001
Magrabi Eye Institute (Yaounde, Cameroon)	3339	2194 (65.7) [64.1–67.3]	1145 (34.3) [32.7–35.9]	< 0.001
**East Africa**	565	353 (62.5) [58.5–66.5]	212 (37.5) [33.5–41.5]	< 0.001
Benjamin Mkapa Hospital (Dodoma, Tanzania)	565	353 (62.5) [58.5–66.5]	212 (37.5) [33.5–41.5]	< 0.001
**Southern Africa**	14 314	10 177 (71.1) [70.4–71.8]	4137 (28.9) [28.2–29.6]	< 0.001
St John Eye Hospital, University of the Witwatersrand (Johannesburg, South Africa)	5074	3532 (69.6) [68.3–70.9]	1542 (30.4) [29.1–31.7]	< 0.001
Sekuru Kaguvi Eye Unit, Paririnyetwa Hospital (Harare, Zimbabwe)	1979	1487 (75.1) [73.2–77.0]	492 (24.9) [23.0–26.8]	< 0.001
Tshwane District Hospital (Tshwane, South Africa)	827	827 (100)	0 (0)	< 0.001
Groote Schuur Hospital, University of Cape Town (Cape Town, South Africa)	3580	2514 (70.2) [68.7–71.7]	1066 (29.8) [31.3–28.3]	< 0.001
Port Elizabeth Provincial Hospital (Gqeberha, South Africa)	1808	1231 (68.1) [65.9–70.2]	577 (31.9) [29.8–34.1]	< 0.001
Dr Agarwal’s Eye Hospital (Maputo, Mozambique)	1046	586 (56.0) [53.0–59.0]	460 (44.0) [41.0–47.0]	< 0.001

^a^One sample proportions test

Of the different sub-specialty surgical categories, cataract and strabismus surgical procedures decreased significantly in each of the regions of sub-Saharan Africa where these procedures are offered (Table 3). West Africa had further reductions in all subspecialty procedures except oncology (*p* = 0.051), and trauma-related (*p* = 0.179); which increased. In addition to cataract and strabismus, Central Africa showed a significant decrease in corneal procedures. All other subspecialties showed a non-significant decrease with glaucoma remaining stable. In East Africa, cornea (*p* = 0.796), glaucoma (*p* = 0.052) and oncology (*p* = 0.564) surgical procedures did not decrease significantly. In Southern Africa, all sub-specialty surgical procedures decreased significantly (Table 3).

**Table 3:** Sub-specialty surgical procedures per region

Region	Total number of surgeries *n*	Pre-pandemic number of surgical procedures *n*, % of total (95%CI)	Pandemic number of surgical procedures *n*, % of total (95%CI)	*p*-value
**Sub-Saharan Africa**	26 357	17 415 (66.1) [65.5–66.7]	8942 (33.9) [33.4–34.5]	< 0.001
Cataract	14 735	10 250 (69.6) [68.8–70.3]	4485 (30.4) [29.7–31.2]	< 0.001
Cornea	1687	1220 (72.3) [70.2–74.5]	467 (27.7) [25.5–29.8]	< 0.001
Glaucoma	1644	954 (58.0) [55.6–60.4]	690 (42.0) [39.6–44.4]	< 0.001
Oncology	886	518 (58.5) [55.2–61.7]	368 (41.5) [38.3–44.8]	< 0.001
Orbital/oculoplastic	926	618 (66.7) [63.7–69.8]	308 (33.3) [30.2–36.3]	< 0.001
Strabismus	564	474 (84.0) [81.0–87.1]	90 (16.0) [12.9–19.0]	< 0.001
Trauma	2034	1070 (52.6) [50.4–54.8]	964 (47.4) [45.2–49.6]	0.019
Vitreoretinal	2958	1770 (59.8) [58.1–61.6]	1188 (40.2) [38.4–41.9]	< 0.001
Other	923	541 (58.6) [55.4–61.8]	382 (41.4) [38.2–44.6]	< 0.001
**West Africa**	7701	4433 (57.6) [56.5–58.7]	3268 (42.4) [41.3–43.5]	< 0.001
Cataract	4270	2469 (57.8) [56.3–59.3]	1801 (42.2) [40.7–43.7]	< 0.001
Cornea	527	374 (70.9) [67.1–74.5]	153 (29.1) [25.5–32.9]	< 0.001
Glaucoma	860	476 (55.3) [52.0–58.7]	384 (44.7) [41.3–48.0]	0.002
Oncology	59	37 (62.7) [50.4–75.1]	22 (37.3) [24.9–49.6]	0.051
Orbital/oculoplastic	146	85 (58.2) [50.2–66.2]	61 (41.8) [33.8–49.8]	0.047
Strabismus	105	42 (40.0) [30.6–49.4]	63 (60.0) [50.6–69.4]	0.040
Trauma	142	63 (44.3) [36.2–52.5]	79 (55.70 [47.5–73.8]	0.179
Vitreoretinal	1152	633 (55.0) [52.1–57.8]	519 (45.0) [42.2–47.9]	< 0.001
Other	440	254 (57.7) [53.1–62.3]	186 (42.3) [37.7–46.9]	0.001
**Central Africa**	3777	2452 (64.9) [63.4–66.4]	1325 (35.1) [33.6–36.6]	< 0.001
Cataract	2752	1860 (67.6) [65.8–69.3]	892 (32.4) [30.7–34.2]	< 0.001
Cornea	309	189 (61.2) [55.7–66.6]	120 (38.8) [33.4–44.3]	< 0.001
Glaucoma	171	85 (49.7) [42.2–57.2]	86 (50.3) [42.8–57.8]	0.939
Oncology	84	46 (54.8) [44.1–65.4]	38 (45.2) [34.6–55.9]	0.383
Orbital/oculoplastic	96	55 (57.3) [47.4–67.2]	41 (42.7) [32.8–52.6]	0.153
Strabismus	52	36 (69.2) [56.7–81.8]	16 (30.8) [18.2–43.3]	0.006
Trauma	193	108 (56.0) [49.0–63.0]	85 (44.0) [37.0–51.0]	0.098
Vitreoretinal	79	46 (58.2) [47.4–69.1]	33 (47.8) [30.9–52.6]	0.144
Other	41	27 (65.9) [51.3–80.4]	14 (34.1) [19.6–48.7]	0.042
**East Africa**	565	353 (62.5) [58.5–66.5]	212 (37.5) [33.5–41.5]	< 0.001
Cataract	489	328 (67.1) [62.9–71.2]	161 (32.9) [37.1–28.8]	< 0.001
Cornea	15	8 (53.3) [28.1–78.6]	7 (46.7) [21.4–71.9]	0.796
Glaucoma	13	10 (76.9) [54.0–99.8]	3 (23.1) [0.2–46.0]	0.052
Oncology	3	1 (33.3) [-20.0–86.7]	2 (66.7) [13.3–120.0]	0.564
Orbital/oculoplastic	19	2 (10.5) [3.3–24.3]	17 (89.5) [75.7–96.7]	< 0.001
Strabismus	0	0	0	
Trauma	20	4 (20.0) [2.4–37.5]	16 (80.0) [62.5–97.6]	0.007
Vitreoretinal	0	0	0	
Other	6	0 (0)	6 (100)	0.014
**Southern Africa**	14 314	10 177 (71.1) [70.4–71.8]	4137 (28.9) [28.2–29.6]	< 0.001
Cataract	7224	5593 (77.4) [76.5–78.4]	1631 (22.6) [21.6–23.5]	< 0.001
Cornea	836	649 (77.6) [74.8–80.5]	187 (22.4) [19.5–25.2]	< 0.001
Glaucoma	600	383 (63.8) [60.0–67.7]	217 (36.2) [33.3–40.0]	< 0.001
Oncology	740	434 (58.7) [55.1–62.2]	306 (41.3) [37.8–44.9]	< 0.001
Orbital/oculoplastic	665	476 (71.6) [68.2–75.0]	189 (28.4) [25.0–31.8]	< 0.001
Strabismus	407	396 (97.3) [95.7–98.8]	11 (2.7) [1.2–4.3]	< 0.001
Trauma	1679	895 (53.3) [50.9–55.7]	784 (46.7) [44.3–49.1]	0.007
Vitreoretinal	1727	1091 (63.2) [60.9–65.4]	636 (36.8) [34.6–39.1]	< 0.001
Other	436	260 (59.6) [55.0–64.2]	176 (40.4) [35.8–45.0]	< 0.001

The IRRs showed a significant decrease in surgical procedures during the pandemic in both the univariate and multivariate analyses (Table 4). On univariate analysis, the number of surgical procedures performed was lower in the first year of the pandemic compared to the year prior to the pandemic by 49% (IRR 0.51, 95% CI 0.41–0.64), 27% (0.73, 0.55–0.99), 46% (0.54, 0.30–0.99), 40% (0.60, 0.39–0.92) and 59% (0.41, 0.29–0.57) in sub-Saharan Africa (4 regions combined), West, Central, East and Southern Africa, respectively. On multivariate analysis, the first and second waves of the pandemic had no further impact on the incidence in sub-Saharan Africa (0.75, 0.51–1.09 and 1.09, 0.75–1.58, respectively), West Africa (0.97, 0.58–1.62 and 1.23, 0.74–2.07, respectively), Central Africa (0.95, 0.33–2.72 and 0.84, 0.29–2.41, respectively) and East Africa (0.52, 0.27–1.04 and 1.60, 0.87–2.93, respectively). However, in Southern Africa, there was a further significant decrease in surgical procedures during the first wave of the pandemic (0.53, 0.30–0.94), whilst this was not the case during the second wave (1.00, 0.57–1.76).

**Table 4:** Univariate and multivariate IRRs of the total surgical procedures in sub-Saharan Africa and each region

	Incidence rate ratio	95% CI	*p* value
**Univariate**			
* Region*			
Sub-Saharan Africa	0.51	0.41–0.64	< 0.001
West Africa	0.73	0.55–0.99	0.045
Central Africa	0.54	0.30–0.99	0.047
East Africa	0.60	0.39–0.92	0.018
Southern Africa	0.41	0.29–0.57	< 0.001
**Multivariate**			
* Sub-Saharan Africa*			
Pandemic	0.54	0.41–0.70	< 0.001
Wave 1	0.75	0.51–1.09	0.136
Wave 2	1.09	0.75–1.58	0.655
*West Africa*			
Pandemic	0.70	0.49–1.01	0.057
Wave 1	0.97	0.58–1.62	0.909
Wave 2	1.23	0.74–2.07	0.422
*Central Africa*			
Pandemic	0.57	0.27–1.20	0.138
Wave 1	0.95	0.33–2.72	0.924
Wave 2	0.84	0.29–2.41	0.748
*East Africa*			
Pandemic	0.58	0.38–0.91	0.017
Wave 1	0.52	0.26–1.04	0.066
Wave 2	1.60	0.86–2.94	0.138
*Southern Africa*			
Pandemic	0.45	0.30–0.67	< 0.001
Wave 1	0.55	0.32–0.98	0.041
Wave 2	1.04	0.59–1.83	0.885

The number of surgical procedures in the different sub-specialty categories in sub-Saharan Africa (4 regions combined) was significantly lower in the pandemic year compared to the pre-pandemic year, except for glaucoma (IRR 0.72, 95% CI 0.52–1.01), oncology (0.71, 0.48–1.05), trauma (0.90, 0.63–1.28) and vitreoretinal (0.67, 0.42–1.08) categories (Table 5). In West Africa, there was a 27% (0.73, 0.54–0.99) and 59% (0.41, 0.27–0.63) reduction in the cataract and cornea surgical procedures performed in the pandemic year, respectively. In Central Africa and East Africa, the only significant reduction was in cataract surgery which decreased by 52% (0.48, 0.23–0.98) and 51% (0.49, 0.39–0.78), respectively. In Southern Africa, except for oncology (0.71, 0.43–1.17) and trauma (0.88, 0.54–1.43), the number of surgical procedures in the different sub-specialty categories decreased significantly in the pandemic year.

**Table 5:** IRRs of each type of surgical procedure per region

Region	IRR	95%CI	*p* value
**Sub-Saharan Africa**			
Cataract	0.44	0.35–0.55	< 0.001
Cornea	0.38	0.29–0.51	< 0.001
Glaucoma	0.72	0.52–1.01	0.061
Oncology	0.71	0.48–1.05	0.087
Orbital/oculoplastic	0.50	0.36–0.69	< 0.001
Strabismus	0.19	0.11–0.32	< 0.001
Trauma	0.90	0.63–1.28	0.562
Vitreoretinal	0.67	0.42–1.08	0.099
Other	0.71	0.49–1.02	0.065
**West Africa**			
Cataract	0.73	0.54–0.99	0.043
Cornea	0.41	0.27–0.63	< 0.001
Glaucoma	0.81	0.46–1.40	0.447
Oncology	0.60	0.31–1.14	0.118
Orbital/oculoplastic	0.72	0.47–1.11	0.133
Strabismus	1.50	0.68–3.30	0.313
Trauma	1.25	0.81–1.92	0.302
Vitreoretinal	0.82	0.39–1.75	0.607
Other	0.73	0.41–1.31	0.295
**Central Africa**			
Cataract	0.48	0.23–0.98	0.045
Cornea	0.64	0.22–1.86	0.407
Glaucoma	1.01	0.46–2.22	0.977
Oncology	0.83	0.51–1.32	0.424
Orbital/oculoplastic	0.75	0.33–1.68	0.48
Strabismus	0.44	0.18–1.11	0.083
Trauma	0.79	0.48–1.28	0.337
Vitreoretinal	0.72	0.08–6.79	0.772
Other	0.51	0.19–1.39	0.192
**East Africa**			
Cataract	0.49	0.31–0.78	0.002
Cornea	0.88	0.16–4.95	0.880
Glaucoma	0.30	0.04–2.17	0.233
Oncology	2.0	0.18–22.1	0.571
Orbital/oculoplastic	8.5	1.42–50.7	0.019
Strabismus	0	0	0
Trauma	4	1.3–12.2	0.015
Vitreoretinal	0	0	0
Other	0	0	0
**Southern Africa**			
Cataract	0.29	0.20–0.42	< 0.001
Cornea	0.29	0.20–0.42	< 0.001
Glaucoma	0.57	0.35–0.91	0.019
Oncology	0.71	0.43–1.17	0.173
Orbital/oculoplastic	0.40	0.26–0.62	< 0.001
Strabismus	0.03	0.01–0.06	< 0.001
Trauma	0.88	0.54–1.43	0.597
Vitreoretinal	0.58	0.34–0.99	0.045
Other	0.68	0.41–1.12	0.126

## Discussion

Our study showed a temporal association between the enforcement of interventions to reduce the spread of the COVID-19 infection and the decreased incidence of ophthalmic surgical procedures during the pandemic year. We observed a decrease in the incidence of surgical procedures (49% reduction), in the four regions of sub-Saharan Africa combined (West, Central, East and Southern), during the first year of the pandemic compared to the year prior. Furthermore, we noted a lower incidence of surgical procedures in West (27% reduction), Central (46% reduction), East (40% reduction) and Southern Africa (59% reduction). We also showed a reduction in the incidence of cataract (56% reduction), cornea (62% reduction), strabismus (81% reduction) and orbital/oculoplastic (50% reduction) surgical procedures in the four regions of sub-Saharan Africa combined. Cataract was the only category that had a lower incidence in the first year of the pandemic in all four regions of sub-Saharan Africa. Regional differences in the impact of the pandemic as well as different implementation of lockdown procedures could account for the variability in the percentage reductions in surgeries.

Our findings of ophthalmic surgical procedures were similar to other non-ophthalmic studies, which reported on lower elective surgical rates (34–55%) during the pandemic year [8, 18, 19]. To mitigate the burden of the COVID-19 pandemic on the healthcare system, healthcare resources were diverted from surgical departments to intensive care facilities by postponing surgical cases, especially elective surgical procedures. Observations of the literature suggest that the disciplines of Ophthalmology and Otorhinolaryngology had a higher reduction (89–94%) in surgical cases than the non-ophthalmic surgical disciplines (34–55%) [8, 11–13, 18, 20]. The higher reduction in the former disciplines may be due to the increased risk of SARS-CoV-2 transmission during these surgical procedures (compared to the non-ophthalmic surgical procedures) and the higher proportion of non-urgent elective surgical procedures. The lower reduction in the other surgical disciplines may also be due to increased treatment of medically necessary time-sensitive surgery (MENTS), such as malignancies. [8, 18]

Our observations of an overall 49% reduction in ophthalmic surgical procedures, in the pandemic year compared to the pre-pandemic year, is in contrast with ophthalmic studies in other regions of the world, which reported on higher reductions in surgical procedures (89–94%) [11–13]. However, these studies reported only or mostly on elective surgical procedures (cataract and cornea), which are reversible causes of visual impairment and blindness, whereas our study reported on a combination of elective and urgent surgical procedures. Additionally, it is possible that less stringent lockdown measures and lower COVID-19 cases in Africa generally (compared with the rest of the world) may have led to a lower reduction in ophthalmic healthcare services, especially ophthalmic surgical procedures during the pandemic year [21–23]. We noted a higher reduction in ophthalmic surgical procedures in Southern Africa (58%) than West (27%), Central (46%) and East (40%) Africa during the pandemic year. Stricter lockdown measures and higher COVID-19 cases in South Africa (which contributed to most of the ophthalmic surgical procedures in the Southern African region) compared to the rest of sub-Saharan Africa probably led to the higher reduction in ophthalmic healthcare services and surgical procedures in the first year of the pandemic in the Southern African region [21, 22]. A higher reduction in elective cataract surgical procedures in Southern Africa (71%) compared to West (27%), Central (52%) and East Africa (51%) supports the temporal relationship between lockdown stringency and the incidence of ophthalmic surgical procedures.

Our findings of a higher reduction in cataract (71%) than glaucoma (43%), vitreoretinal (42%), oncology (29%) and trauma (12%) surgical procedures in Southern Africa, albeit the latter two not being significant, were similar to a study from the USA, which reported no cataract surgical procedures and higher proportions of glaucoma, vitreoretinal, oncology and trauma surgical procedures in the first year of the pandemic compared to a similar period in the year prior to the pandemic [12]. Overall in sub-Saharan Africa, we documented similar findings, albeit all four latter categories not being significant. Furthermore, cataract surgical procedures decreased significantly in each of the four regions (West, Central, East and Southern Africa); however, oncology, including exenterations and enucleations for tumours, and trauma surgical procedures did not decrease significantly in each of the regions. Cataract surgical procedures can be delayed without causing permanent visual impairment and blindness, and hence the significant reduction of these procedures. However, glaucoma, vitreoretinal, oncology and trauma surgical procedures are usually medically necessary time-sensitive (MENTS) surgical procedures, which if not addressed timeously can result in irreversible visual impairment and blindness. A non-ophthalmic orthopaedic study also reported a higher reduction in elective surgical procedures (85–100%) than trauma and emergency surgical procedures (40%), including oncology in the pandemic year compared to a similar period in the pre-pandemic year [18].

Our observations of a decrease in the incidence of trauma in Central and Southern Africa (albeit not being significant) contrasted with the findings in West and East Africa, which had an increase in the incidence of trauma. The increase incidence in West Africa was similar to a multicentre study from Nigeria, which reported an increase in trauma, especially amongst children [14]. However, in our study, the absolute number of trauma cases was small in East and West Africa. These differences are most likely due to social and societal differences between the regions.

Limitations of our study include, due to its retrospective design, being unable to determine whether changes in health-seeking behaviour, such as reluctance to visit ophthalmic departments, could have contributed to the reduced numbers of surgical procedures, especially in the pandemic year. However, several other studies have also reported on the disruption of ophthalmic surgical services during the pandemic year [11–13]. Furthermore, we did not observe a significant change in trauma surgical procedures in the pandemic year compared to the pre-pandemic year. Since there is a low threshold for trauma cases, especially penetrating injuries, requiring surgery, our data indicate that health-seeking behaviour may not have been considerably affected in the first year of the pandemic. Another limitation of our study was that data were extracted manually from the theatre surgical records, and entered into data sheets; thus, it is possible that data could have been incorrectly extracted and entered in certain instances. However, a rigorous process of collecting, checking and verifying the data was undertaken by two investigators per site and further verified by investigators at the primary site. A further limitation is the limited data from East Africa, where only one hospital participated in the study. Consequently, this region was underrepresented and therefore ophthalmic surgical data from this hospital may not be a true representation of the region. In addition, we assessed the first year of the pandemic and it would be worthwhile looking at the following years and waves to get a clearer picture of the impact of the pandemic. Our study mainly focuses on the early impact.

## Conclusion

This study is the first multicentre sub-Saharan African study which provides the most comprehensive insight into the effects of the COVID-19 pandemic on the epidemiology of ophthalmic surgical procedures in sub-Saharan Africa. Studying the effects of the pandemic on surgical numbers will hopefully enable a better understanding of which ophthalmology surgical subspecialties were mostly affected, thus enabling a better and more tailored recovery plan after the pandemic.

## Data Availability

The datasets used and/or analysed during the current study are available from the corresponding author on reasonable request.

## Appendix A

**Table:**

Hospital	Dates of pre–pandemic year	Dates of pandemic year	Months of first wave	Months of second wave
**West Africa**				
St Thomas Eye Hospital (Accra, Ghana)	30/03/2019–29/03/2020	30/03/2020–29/03/2021	May 2020 to July 2020	October 2020–December 2020
Federal Medical Centre (Asaba, Nigeria)	30/03/2019–29/03/2020	30/03/2020–29/03/2021	May 2020 to July 2020	October 2020–December 2020
Enugu State University Teaching Hospital (Enugu, Nigeria)	30/03/2019–29/03/2020	30/03/2020–29/03/2021	May 2020 to July 2020	October 2020–December 2020
Eye Foundations Hospital (Lagos, Nigeria)	30/03/2019–29/03/2020	30/03/2020–29/03/2021	May 2020 to July 2020	October 2020–December 2020
The Eye Specialists Hospital (Enugu, Nigeria)	30/03/2019–29/03/2020	30/03/2020–29/03/2021	May 2020 to July 2020	October 2020–December 2020
University of Ilorin Teaching Hospital (Ilorin, Nigeria)	30/03/2019–29/03/2020	30/03/2020–29/03/2021	May 2020 to July 2020	October 2020–December 2020
Usmanu Danfodiyo University Hospital (Sekoto, Nigeria)	30/03/2019–29/03/2020	30/03/2020–29/03/2021	May 2020 to July 2020	October 2020–December 2020
**Central Africa**				
Yaounde Gynaeco–obstetric and paediatric hospital (Yaounde, Cameroon)	18/03/2019–17/03/2020	18/03/2020–17/03/2021	May 2020 to July 2020	March 2021 to May 2021^a^
Yaounde Central Hospital (Yaounde, Cameroon)	18/03/2019–17/03/2020	18/03/2020–17/03/2021	May 2020 to July 2020	March 2021 to May 2021^a^
Magrabi Eye Institute (Yaounde, Cameroon)	18/03/2019–17/03/2020	18/03/2020–17/03/2021	May 2020 to July 2020	March 2021 to May 2021^a^
**East Africa**				
Benjamin Mkapa Hospital (Dodoma, Tanzania)	29/03/2019–28/03/2020	29/03/2020–28/03/2021	June to August 2020	November 2020 to January 2021^b^
**Southern Africa**				
St John Eye Hospital, University of the Witwatersrand (Johannesburg, South Africa)	27/03/2019–26/03/2021	27/03/2020–26/03/2021	June to August 2020	November 2020 to January 2021
Sekuru Kaguvi Eye Unit, Paririnyetwa Hospital (Harare, Zimbabwe)	30/03/2019–29/03/2021	30/03/2020–29/03/2021	June to August 2020	December 2020 to January 2021
Tshwane District Hospital (Tshwane, South Africa)	27/03/2019–26/03/2021	27/03/2020–26/03/2021	June to August 2020	November 2020 to January 2021
Groote Schuur Hospital, University of Cape Town (Cape Town, South Africa)	27/03/2019–26/03/2021	27/03/2020–26/03/2021	June to August 2020	November 2020 to January 2021
Port Elizabeth Provincial Hospital (Gqeberha, South Africa)	27/03/2019–26/03/2021	27/03/2020–26/03/2021	June to August 2020	November 2020 to January 2021
Dr Agarwal’s Eye Hospital (Maputo, Mozambique)	01/04/2019–31/03/2020	01/04/2020–31/03/2021	June to August 2020	November 2020 to January 2021

^a^Only March 2021 data was included as the April and May data fell out of the study period.

^b^Data extrapolated as no official data exists.
